# Supplementary material for: Optomechanical ring resonator for efficient microwave-optical frequency conversion
Source: Nat Commun. 2023 Nov 21;14:7594. doi: 10.1038/s41467-023-43393-x (PMC10663453; doi:10.1038/s41467-023-43393-x)
Supplement: Supplementary file 1 — Supplementary Information [file 41467_2023_43393_MOESM1_ESM.pdf]

# Supplementary Notes for optomechanical ring resonator for efficient microwave-optical frequency conversion

I-Tung Chen,[1] Bingzhao Li,[1] Seokhyeong Lee,[1] Srivatsa Chakravarthi,[2] Kai-Mei Fu,[1,2,3] and Mo Li[1,2,*]

[1]*Department of Electrical and Computer Engineering, University of Washington, Seattle, WA 98115, USA*

[2]*Department of Physics, University of Washington, Seattle, WA 98115, USA*

[3]*Physical Sciences Division, Pacific Northwest National Laboratory, Richland, Washington, 99352 USA*

### Supplementary Note 1. COUPLED-MODE THEORY (CMT) ANALYSIS

We employ the coupled-mode theory (CMT) to analyze the OMR system. The electric field propagating in the OMR experiences a perturbed dielectric medium ($\Delta\varepsilon$) hence induces a perturbed polarization field:

$$\Delta\mathbf{P} = \Delta\varepsilon(x,y,z)\mathbf{E}_i(x,y)e^{i(\omega t-\beta_i z)}.$$

where $\beta_i$ is the propagating wavevector of the i-th mode, $E_i(x,y)$ is the normal mode electric field of the i-th mode. The mechanical perturbation of the permittivity $\Delta\varepsilon$ consists of contributions from the moving boundary effect[1] and the photoelastic effect[2] and can be described as

$$\Delta\varepsilon = \delta\varepsilon\cdot\mathbf{u} = -\underbrace{\mathbf{u}(x,y)\cdot\mathbf{n}(x,y)(\Delta\varepsilon'|\mathbf{E}_{\parallel}(x,y)|^2 - \Delta\varepsilon'^{-1}|\mathbf{D}_{\perp}|^2)}_{\text{Moving boundary effect}} + \underbrace{\frac{\varepsilon\cdot p\mathbf{S}\cdot\varepsilon}{\varepsilon_0}}_{\text{Photoelastic effect}}$$

where $\mathbf{n}$ is the normal vector pointing from the dielectric 1 to dielectric 2; $\Delta\varepsilon' = \varepsilon_2 - \varepsilon_1$ and $\Delta\varepsilon'^{-1} = \varepsilon_2^{-1} - \varepsilon_1^{-1}$; $\mathbf{E}_{\parallel}$ and $\mathbf{D}_{\perp}$ are the tangential electric field and perpendicular displacement field at the dielectric boundary; p is the photoelastic tensor and $\mathbf{S}$ is the strain tensor. The mechanical displacement field of the acoustic wave is:

$$\mathbf{u}(x,y,z,t) = \mathbf{u}(x,y)e^{i(\Omega t-Kz)} + \text{c.c.}$$

where $\Omega$ is the acoustic frequency, $K$ is the acoustic wavevector. The optical field is expressed as:

$$\mathbf{E}_i(x,y,z,t) = A_i\mathbf{e}_i(x,y)e^{i(\omega t-\beta_i z)} + \text{c.c.}$$

where $A_i$ is the electric field amplitude which is normalized to equal to the optical power $P_i$. Here, we only consider two relevant modes: $i=0$ for the $TE_0$ mode and $i=2$ for the $TE_2$. Defining the phase mismatch $\Delta\beta = \beta_0 - \beta_2 - K \neq 0$, the mode coupling equations are:

$$\frac{d}{dz}A_2(z) = -iG_{20}A_0(z)e^{-i\Delta\beta z} \tag{SI.1}$$

where the total coupling coefficient $G_{20}$ is given by:

$$G_{20} = -\frac{\omega_2}{2}\frac{\int dA\mathbf{E}_2^*\cdot\delta\varepsilon\cdot\mathbf{u}(x,y)\cdot\mathbf{E}_0}{P_2}.$$

And,

$$\frac{d}{dz}A_0(z) = -iG_{02}A_2(z)e^{i\Delta\beta z} \tag{SI.2}$$

where

$$G_{02} = -\frac{\omega_0}{2}\frac{\int dA\mathbf{E}_0^*\cdot\delta\varepsilon\cdot\mathbf{u}^*(x,y)\cdot\mathbf{E}_2}{P_0}.$$

* Corresponding author: moli96@uw.edu

Since the coupling rate is proportional to the square root of the phonon flux $\sqrt{\Phi} = \sqrt{P_a/\hbar\Omega}$, we define the flux normalized coupling rate $g \equiv G/\sqrt{\Phi} = G_{20}/\sqrt{\Phi} = G_{02}/\sqrt{\Phi}$. The solutions of $A_0(z)$ and $A_2(z)$ in the general case when $\Delta\beta \neq 0$ are:

$$A_0(z) = A_0(0)[\cos(sz) - \frac{i\Delta\beta}{2}\frac{\sin(sz)}{s}]e^{i\Delta\beta z/2} \tag{SI.3}$$

$$A_2(z) = A_0(0)[-iG\frac{\sin(sz)}{s}]e^{i\Delta\beta z/2}. \tag{SI.4}$$

where $A_0$ and $A_2$ are the normalized amplitudes of the two modes that are traveling in the OMR, and $s = \sqrt{G^2 + (\Delta\beta/2)^2} = \sqrt{\frac{g^2}{\hbar\Omega}P_a + (\Delta\beta/2)^2}$ and the internal power conversion efficiency is when $z = \pi D$, where D is the OMR diameter:

$$\eta_i(P_a, z = \pi D) = |\frac{A_2(P_a, z = \pi D)}{A_0(0)}|^2.$$

$\eta_i$ reaches maximum when $s\pi D = \pi/2$, so $s = 1/(2D)$. The corresponding acoustic power $P_a$ is defined as $P_{\pi/2}$. When the intra-cavity $TE_0$ is circulating the OMR, the optomechanical coupling to the $TE_2$ mode can be viewed as additional attenuation and change the waveguide-ring coupling condition that is dependent on the acoustic power, given by $b(P_a) = A_0(z)/A_0(0)$. Thus, the total $TE_0$ mode attenuation coefficient to be $a(P_a) = a_0 + b(P_a)$, where $a_0 = e^{-\alpha\pi D}$, with $\alpha$ being the propagation loss with a unit of $mm^{-1}$. With this modification, the output field amplitude at the drop port can be described with the standard ring resonator model using the transmission coefficient $t_0$, $t_2$ and the cross-coupling coefficient $\kappa_0$, $\kappa_2$, as shown in the Fig. S1. Combining the ring resonator model and equation SI.3, the drop port amplitude can be expressed as

$$A_{TE_0-drop}(P_a) = A_{TE_0-in}\frac{\kappa_0\kappa_2 a(P_a)e^{-i\phi/2}}{1 - t_0t_2a(P_a)e^{-i\phi}}$$

where $\phi$ is the round trip phase. The $TE_0$ intensity at the resonance condition ($\phi = 2\pi$):

$$A^2_{TE_0-drop}(P_a) = [A_{TE_0-in}\frac{\kappa_0\kappa_2 a(P_a)}{1 - t_0t_2a(P_a)}]^2 \tag{SI.5}$$

The $TE_2$ mode output intensity can be expressed as

$$A^2_{TE_2-drop} = [A_{TE_0-drop}(P_a)]^2\eta(P_a). \tag{SI.6}$$

We use this relation to fit the results in Fig. 4 d and e in the main text to extract the value of $P_{\pi/2}$.

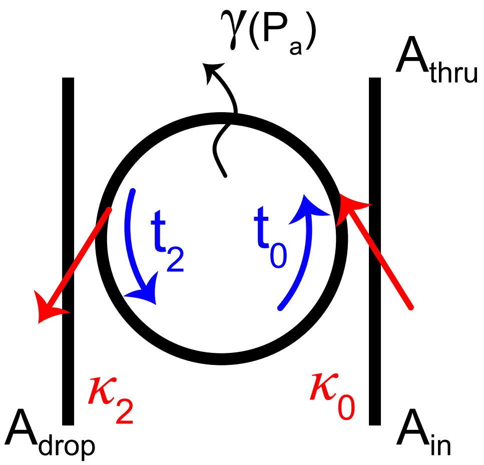


FIG. S1. **CMT model of the OMR.**

## Supplementary Note 2. OPTOMECHANICAL CONVERSION EFFICIENCY CALCULATION

The internal optomechanical internal conversion efficiency is defined as

$$\eta_i = \frac{P_{TE_2}}{P_{TE_0}}$$

where $P_{TE_2}$ is the optomechanical converted $\mathrm{TE}_2$ mode power inside the OMR, and $P_{TE_0}$ is the power of $\mathrm{TE}_0$ mode that coupled into OMR. $P_{TE_0}$ is known from the input laser power, the grating coupler efficiency, and the measured transmission of the $\mathrm{TE}_0$ mode in the through port. So, to calculate $\eta_i$, we need to obtain the $P_{TE_2}$. We use two methods to obtain $P_{TE_2}$. The first method is by bookkeeping the optical power all the way from the RSA measured electrical power to the optical power inside the OMR. The second method is to use the fitting of equation SI.5 & SI.6 to obtain the intra-cavity field at $A_2(gD\pi\sqrt{P_{\pi/2}} = \pi/2)$ and calculated $P_{TE_2}$ to obtain $\eta_i$. We will describe both methods in this section.

### The Bookkeeping method

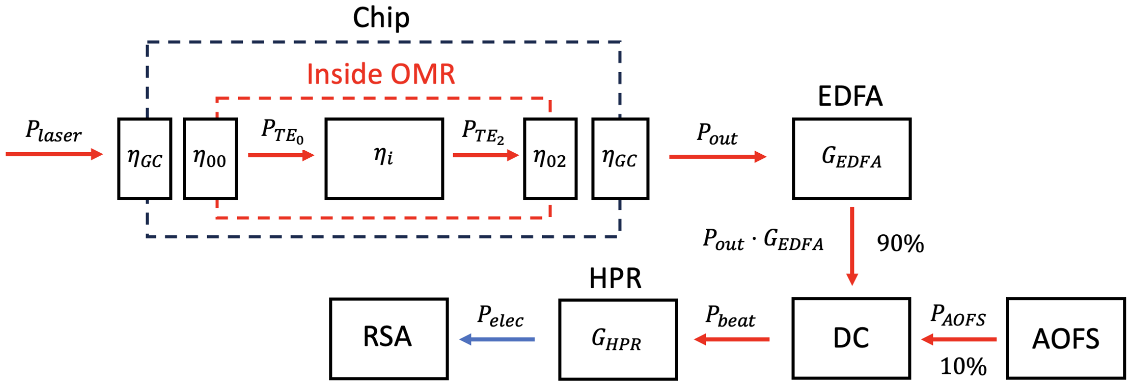


FIG. S2. $TE_0$**-to-**$TE_2$ **optomechanical conversion efficiency calculation flowchart.** The red arrows represent the path of the optical signal, and the blue arrow represents the electrical signal. The input laser power $P_{\mathrm{laser}}$, is coupled to the OMR through a grating coupler with coupling efficiency $\eta_{GC}$ and the waveguide-to-OMR coupling efficiency $\eta_{00}$. The input $TE_0$ mode is then converted to $TE_2$ mode at the OMR with internal efficiency $\eta_i$. The $TE_2$ mode is then coupled out of the OMR with efficiency $\eta_{02}$ and out of the chip from the grating coupler with efficiency $\eta_{GC}$. The output $TE_2$ optical power is labeled as $P_{\mathrm{out}}$. $P_{\mathrm{out}}$ is then amplified by EDFA with gain $G_{\mathrm{EDFA}}$ and beats with the AOFS in a directional coupler (DC). The beating optical signal $P_{\mathrm{beat}}$ is finally detected by the high-speed photoreceiver (HPR) and converted to electrical signal $P_{\mathrm{elec}}$ which is measured by the RSA.

The $\mathrm{TE}_2$ optical power output from the OMR is first amplified by an erbium-doped fiber amplifier (EDFA), and combined with the acousto-optic frequency shifter (AOFS) reference signal, then converted to electrical power by the high-speed photoreceiver (HPR), and finally measured by the RSA, as shown in Fig. S2. We start the bookkeeping calculation from the RSA measured power. The conversion of the beating signal optical power $P_{\mathrm{beat}}$ to electrical power $P_{elec}$ via HPR can be expressed as

$$P_{\mathrm{elec}} = (G_{\mathrm{HPR}} \cdot P_{\mathrm{beat}})^2 / R_0$$

where the gain of the HPR is $G_{\mathrm{HPR}} = 500$ V/W and the characteristic impedance is $R_0 = 50$ Ohm. And the optical power $P_{\mathrm{beat}}$ is the beating signal of $P_{\mathrm{out}}$ and $P_{\mathrm{AOFS}}$, which can be expressed as

$$P_{\mathrm{beat}} = \sqrt{0.9 P_{\mathrm{out}} \cdot G_{\mathrm{EDFA}} \cdot 0.1 P_{\mathrm{AOFS}}}$$

where the gain from EDFA is $G_{\mathrm{EDFA}} = 14.77$ dB, the $P_{\mathrm{AOFS}} = 0.038$ mW. $G_{\mathrm{EDFA}}$ had been calibrated using a power meter at 1550 nm. The pre-factors 0.9 and 0.1 are the table-top directional coupler's transmission coefficient that is used to combine the AOFS signal and the device output signal.

$$P_{\mathrm{out}} = \frac{P_{\mathrm{elec}} R_0 / G_{\mathrm{HPR}}^2}{G_{\mathrm{EDFA}} \cdot 0.9 \cdot 0.1 \cdot P_{AOFS}}$$

To calculate the TE2 mode power inside the OMR $P_{TE2}$, we consider the grating coupler efficiency $\eta_{\mathrm{GC}}$ and the $\mathrm{TE}_2$ out-couple efficiency $\eta_{02}$ to reach:

$$P_{TE2} = \frac{P_{\mathrm{out}}}{\eta_{GC}\eta_{02}} = \frac{P_{\mathrm{elec}} R_0 / G_{\mathrm{HPR}}^2}{\eta_{GC}\eta_{02} G_{\mathrm{EDFA}} \cdot 0.9 \cdot 0.1 \cdot P_{AOFS}}$$

where $\eta_{GC} = 3.0\%$ and $\eta_{02} = 3.0\%$, respectively. On the input side, the $\mathrm{TE}_0$ power in the ring can be calculated as

$$P_{TE0} = P_{\mathrm{laser}} \cdot \eta_{GC} \cdot \eta_{00}$$

where $\eta_{GC} = 3.0\%$, and $\eta_{00} = 90.0\%$. Finally, the system's total conversion efficiency is defined as

$$\eta_{\mathrm{tot}} = \frac{P_{\mathrm{out}}}{P_{\mathrm{laser}}}$$

where $P_{\mathrm{out}}$ is the output power from the device and $P_{\mathrm{laser}}$ is the laser input power. Using the above bookkeeping procedure, we calculate the internal conversion efficiency to be $\eta_i = 2.1\%$ and the total device conversion efficiency to be $\eta_{tot} = 0.57 \times 10^{-6}$, when the $P_{\pi/2} = 1.6$ mW acoustic pump power is used.

## The CMT model fitting method

The optomechanical mode conversion efficiency $\eta_i$ can be extracted by fitting equation (SI.5) and (SI.6) to the data. We fit $P_{02}(\Omega - \delta)$ in Fig. 4e in main text using equation (SI.6) with the parameter listed in table S1. The measured data $P_{02}(\delta)$ in Fig. 4(d) is only a reference signal that is proportional to $A^2_{TE_0-drop}(P_a)$, therefore, we plot the fitting in Fig. 4d by considering the HPR conversion gain, the gain of EDFA, and the AOFS signal power. At the critical acoustic power $P_a = P_{\pi/2} = 1.6$ mW, we determine $A^2_{TE_0-drop} = 2.94$ nW, and $A^2_{2-drop} = 0.42$ nW. The resulting $\eta_i = (A_{TE_2-drop}/A_{TE_0-drop})^2 = (6.9 \pm 1.0) \times 10^{-2}$. The fixed parameters are the known values in our system, including the coupling coefficient from bus waveguide to ring, and the loss introduced by optomechanical mode conversion. Therefore, the conversion efficiencies determined by this approach is higher than the bookkeeping method but on the same order of magnitude.

The acoustic wave in the OMR is treated with similar ring resonator theory. We extract the waveguide-OMR acoustic coupling coefficient $\kappa_a$ and the acoustic attenuation coefficient $b_a$ by fitting the results in Fig. 2d.

| Fixed parameters | | | | | Fit parameters | |
|---|---|---|---|---|---|---|
| $A_{0-in}$ ($\sqrt{W}$) | $\kappa_0$ | $\kappa_2$ | $\kappa_a$ | $b_a$ | $g/\sqrt{\hbar\Omega}$ ($\mathrm{mm}^{-1}\sqrt{\mathrm{W}^{-1}}$) | $\Delta\beta$ ($\mu m^{-1}$) |
| 0.02 | 0.95 | 0.17 | 0.80 | 0.86 | 230 | 0.079 |

TABLE S1. Fitting parameters that are used to calculate $g$

We can also extract the optomechanical coupling coefficient $g$ and the phase mismatch $\Delta\beta$ from the CMT fitting. Using the calculated optomechanical coefficient, we can calculate the conversion power for a unity conversion efficiency when $\Delta\beta = 0$ using

$$\sqrt{P_{\pi/2}}\,|_{\Delta\beta=0} = \frac{\sqrt{\hbar\Omega}}{2gD} = 0.01\sqrt{W}$$

the resulted power to achieve unity conversion efficiency when $\Delta\beta = 0$ is 0.1 mW.

## Finite Element Method Simulations

Here we discuss the simulation of the presented optomechanical system and calculate the optomechanical coupling coefficient $g_0$. As stated above, the optomechanical coupling is facilitated through traveling waves in the OMR system, which is different from the previous standing-wave systems. Therefore, instead of simulating the whole OMR system, we simulate the acoustic and optical mode profile of a cross-section of the waveguide, as shown in the main text Fig. 1. We use finite element method (FEM) in COMSOL 5.6 to simulate the cross-sectional mode profile and calculate $g_0$ from the moving boundary (MB) and the photoelastic (PE) contribution using the following expressions

$$\frac{g_0}{\sqrt{\hbar\Omega}} = \frac{G_{MB}}{\sqrt{P_a}} + \frac{G_{PE}}{\sqrt{P_a}} \text{ unit:} \frac{1}{\mathrm{mm}\sqrt{\mathrm{W}}}$$

where $G_{MB}$ and $G_{PM}$ are the optomechanical coupling coefficient contributed by the MB and PE effect, respectively. The following table provides the corresponding simulated value and the calculated $g_0$. The calculated

$$g_0/\sqrt{\hbar\Omega} = 574.3\ mm^{-1}\sqrt{W^{-1}}$$

which is about 2.5 times larger than the measured $g_0$ from data. The simulated $g_0/\sqrt{\hbar\Omega}$ has a $P_{\pi/2} = 0.02$ mW with OMR diameter D=200 $\mu m$. We attribute the discrepancy between the simulatio and the experiment to the refractive index difference between the simulated GaP and the BGaP used in the experiment.

| $\omega_0/2\pi$ (THz) | $\omega_m/2\pi$ (GHz) | $G_{MB}/\sqrt{P_a}$ $(mm^{-1}\sqrt{W^{-1}})$ | $G_{PE}/\sqrt{P_a}$ $(mm^{-1}\sqrt{W^{-1}})$ | $P_a$ (W) |
|---|---|---|---|---|
| 190.80 | 2.56 | -7.9 | 582.2 | $3.8 \times 10^{-3}$ |

TABLE S2. Parameters from FEM simulation

## Supplementary Note 3. MICROWAVE-TO-OPTICAL TRANSDUCTION EFFICIENCY

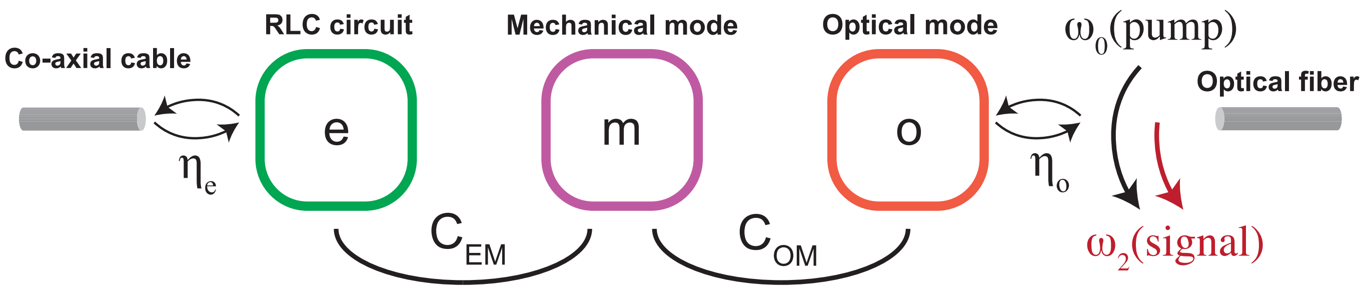


FIG. S3. **General schematic for microwave-to-optical conversion.**

A general schematic of the microwave-to-optical conversion of our device is shown in Fig. S3. Here, the microwave-to-optical conversion is a two-stage process, where the microwave signal is coupled to the mechanical resonator via electromechanical coupling with a coupling rate $g_{EM}$, and the mechanical resonator is coupled to the optical resonator via optomechanical coupling with a coupling rate $g_{OM}$. In this section, we will characterize key parameters related to the microwave-to-optical transduction. The following calculations are based on the work of Wu et al. [3], Han et al. [4] and Aspelmeyer et al. [5].

### A. Calculating Cooperativities

A general form of the cooperativity between coupled resonators can be expressed as:

$$C_{ij} = \frac{4g_{ij}^2}{\kappa_i \kappa_j}$$

where index i, j = (O, M, E) refers to optical, mechanical, and electrical domains, respectively. And $\kappa_i(\kappa_j)$ is the damping rate in the $i(j)$ domain. $C_{EM}$ is the cooperativity between the microwave and the mechanical resonators, and $C_{OM}$ is the cooperativity between the optical and the mechanical resonators.

#### *1. Calculating $C_{EM}$*

First, we calculate the $C_{EM}$ using the expression

$$C_{EM} = \frac{4g_{EM}^2}{\gamma_m \kappa_e}$$

where $g_{EM}$ is the electromechanical coupling rate, $\gamma_m$ is the mechanical energy loss rate (unit: $\text{Hz} \cdot 2\pi$), and $\kappa_e$ is the electrical decay rate (FWHM). The $g_{EM}$ (unit: $\text{Hz} \cdot 2\pi$) can be extracted from the modeled effective circuit in [3] using

$$g_{EM} = \frac{\sqrt{k_T^2}\omega_m}{2}$$
$$k_T^2 = \frac{C_m}{C_m + C_p + C_T}$$

where $k_T^2$ is the reduced piezoelectric coupling strength and $\omega_m = \omega_{LC}$ is the resonance frequency of the RF reflection spectrum. In our model, $C_T$ is absorbed into $C_p$ since the two capacitors are connected in parallel. The modified Butterworth Van Dyke (mBVD) model of the piezoelectric circuit for our system is shown in Fig. S4(a). The electrical decay rate $k_e$ can be extracted from the FWHM of the RF reflection spectrum at resonance frequency, which is shown in Fig. S4(c).

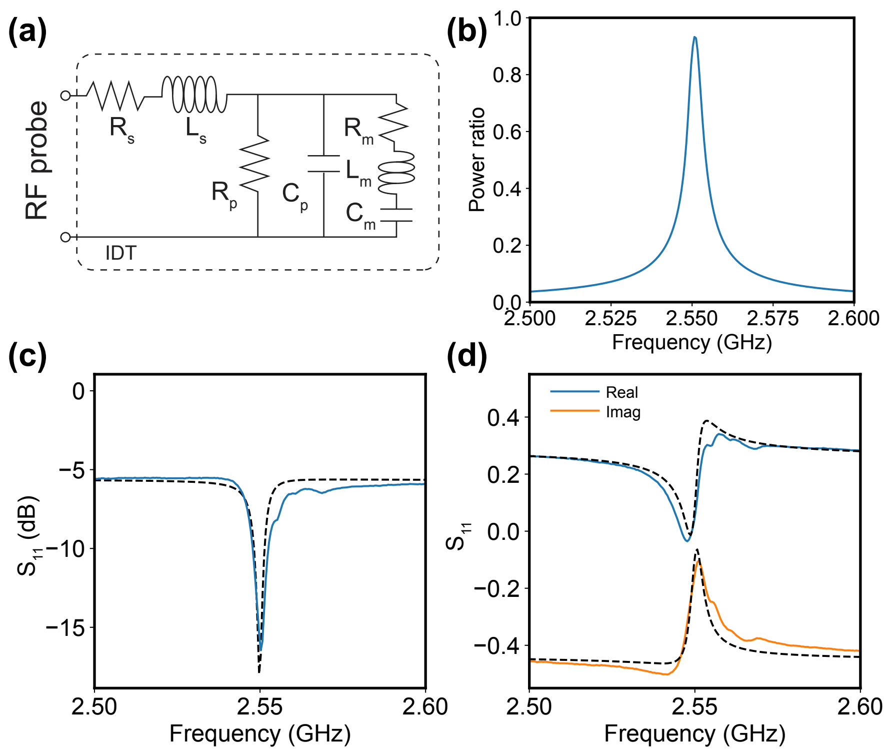


FIG. S4. **Fitting result of the mBVD model at** $4\ K$ **(a)** The mBVD model that is used to fit the RF reflection spectrum ($S_{11}$). **(b)** The ratio of electrical power dissipated on the mechanical lumped element ($R_m$), which corresponds to the power converted to the mechanical power. **(c)** The measured $S_{11}$ spectrum (solid blue line) and the mBVD model fitting (black dashed line). **(d)** The real and imaginary part of the $S_{11}$ spectrum of the measured IDT. The dashed lines are the mBVD fitting.

We use the mBVD model to fit the $S_{11}$ data to extract $C_p$ and $C_m$. Fig. S4(c)(d) show the fitting result of the mBVD model. The fitted $C_p = 0.82\ pF$ and $C_m = 0.0013\ pF$. The fitting result is consistent with the measured data, and we can use the extracted capacitance to calculate

$$
\begin{aligned}
k_T^2 &= \frac{C_m}{C_m + C_p + 0} = \frac{0.0013\ pF}{0.82\ pF + 0.0013\ pF} \sim 1.582 \times 10^{-3} \\
\omega_m/2\pi &= 2.556\ (\text{GHz}) \\
g_{EM} &= \frac{\sqrt{k_T^2}\omega_m}{2} = 319.5\ (\text{Mrad/s}) \\
\kappa_e &= 15\ \text{MHz} \\
\gamma_m &= 1.1\ \text{MHz}
\end{aligned}
$$

and we can calculate the $C_{EM}$ as:

$$
C_{EM} = \frac{4g_{EM}^2}{\gamma_m \kappa_e} = \frac{4 \cdot (319.5 \times 10^6)^2}{15 \times 10^6 \cdot 1.1 \times 10^6 (2\pi)^2} = 626
$$

### 2. *Calculating* $C_{OM}$

Optomechanical cooperativity can be calculated as:

$$
C_{OM} = \frac{4g_{OM}^2}{\gamma_m \kappa_o}
$$

where $g_{OM}$ is the pump-enhanced coupling rate $g_{OM} = g_0\sqrt{n_{\text{phot}}}$, and $n_{\text{phot}}$ is the intra-cavity photon number. Single photon optomechanical coupling rate $g_0$ can be simulated using finite element analysis (FEA). Experimentally, $g_0$ can

be extracted from the measured data and $\sqrt{n_{\text{phot}}}$ can be calculated from the optical pump power. $\gamma_m$ can be extracted from the acoustic quality factor, and $\kappa_0$ can be extracted from the optical quality factor.

The phonon-flux normalized optomechanical coupling rate measured from the data $g_{\text{extract}}/\sqrt{\hbar\Omega} = 230(\frac{1}{mm\sqrt{W}})$, and we can calculate the pump-enhanced coupling rate as:

$$g_{OM} = g_0\sqrt{N_{phot}} = (\frac{g_{\text{extract}}}{\sqrt{\hbar\Omega}}\sqrt{P_a} \times \pi D) \times \sqrt{N_{phot}}$$

where $D$ is the diameter of the OM ring, $P_a$ is the RF pump power, and $\Omega$ is the frequency of the $L_2$ mode. We first perform the following calculations.

$$\begin{aligned} g_0\sqrt{N_{phot}} &= (\frac{g_{\text{extract}}}{\sqrt{\hbar\Omega}} \times \sqrt{1.5 \cdot 10^{-3}}200 \cdot 10^{-3}\pi)\sqrt{N_{phot}} \\ &= 5.7\sqrt{N_{phot}} \\ &= 293 \cdot 10^6 = g_{OM} \\ \gamma_m &= 1.1 \text{ MHz} \\ \kappa_o &= 2.4 \text{ GHz} \end{aligned}$$

and we can calculate the $C_{OM}$ as:

$$C_{OM} = \frac{4g_{OM}^2}{\gamma_m k_e} = \frac{4 \cdot (293 \cdot 10^6)^2}{1.1 \cdot 10^6 \times 2.4 \cdot 10^9 (2\pi)^2} = 3.35$$

### B. Added noise

Following the treatment in reference [3], the added noise $N$ arises from two main contributions in our platform: optical noise contributed by the Stokes scattering $N_o$ and the thermomechanical noise from the pumped phonon $N_m$. The optical added noise can be expressed as:

$$N_o = \frac{1}{\eta_e}\frac{C_{OM}\mathcal{L}_-^2}{C_{EM}}$$

where the electrical coupling efficiency is given by $\eta_e = Z_{tx}/(Z_{tx} + R_s)$. $Z_{tx}$ is the characteristic impedance and $R_s$ is the serial resistance of the mBVD model. The optical-cavity Lorentzian sideband amplitudes are expressed as

$$\mathcal{L}_\pm^2 = \frac{(\kappa_o/2)^2}{(\kappa_o/2)^2 + (\Delta \pm \omega_m)^2},$$

where $\Delta = \omega_{\text{pump}} - \omega_0$ is the laser detuning from cavity resonance, $\kappa_0$ is the optical mode decay rate, $\omega_m$ is the acoustic resonance frequency. In our device, the calculated Stokes sideband amplitude $\mathcal{L}_-^2 = 0.18$. We can use the information calculated so far to calculate optical added noise as:

$$N_o = \frac{1}{0.72}\frac{3.35 \cdot (0.18)}{626} = 0.0013$$

The mechanical thermal noise can be expressed as:

$$N_m = \frac{1}{\eta_e}\frac{n_m}{C_{EM}}$$

where $n_m(\omega) = [e^{\hbar\omega/(k_B T)} - 1]^{-1}$ is given by the Bose-Einstein distribution and $\omega = \omega_m$. We can calculate the mechanical thermal noise as:

$$N_m = \frac{1}{0.72}\frac{33}{626} = 0.07$$

And the total added noise is the sum of the two contributions:

$$N_{add} = N_o + N_m = 0.0713$$

## Supplementary Note 4. EXTRACTING ELECTROMECHANICAL CONVERSION EFFICIENCY AND CHARACTERIZING THE ACOUSTIC TRANSMISSION SIGNAL $|S_{21}|$

### mBVD model and electromechanical conversion efficiency of the IDT

The IDT is modeled using the mBVD model shown in Fig. S4(a). The resistor $R_s$ accounts for the total serial resistance between the RF probe and the IDT fingers, the inductor $L_s$ accounts for the inductive background signal in the cryogenic environment. The shunt resistor $R_p$ and the capacitor $C_p$ account for the effective leakage resistance and electrode capacitance between the IDT fingers, respectively. The electromechanical response of the transducer is modeled with a complex and frequency-dependent admittance $Y_m = R_m + 1/j\omega C_m + j\omega L$, as generalized from the mBVD model [6]. To extract the IDT conversion efficiency, we use the mBVD model to obtain the power load $P_m$ on the mechanical lumped elements $Y_m$. We can simplify the mBVD circuit as the following.

$$
\begin{aligned}
&Z_{\text{total}} = Z_s + Z_{\text{load}},\\
&Z_s = R_s + j\omega L_s,\\
&Z_{\text{load}} = \left(\frac{1}{Z_p} + \sum_N Y_m^{(N)}(\omega)\right)^{-1},\\
&Z_p = \frac{1}{j\omega C_p + 1/R_p}
\end{aligned}
$$

and

$$
Y_m(\omega) = \frac{1}{Z_m} = \frac{1}{R_m + j\omega L_m + 1/j\omega C_m}. \tag{SI.7}
$$

The total effective impedance $Z_{\text{total}}$ can be calculated from the measured $S_{11}$ using the following expression

$$
Z_{\text{total}} = R_{char} \times \frac{1 + \mathbb{C}(S11(\omega))}{1 - \mathbb{C}(S11(\omega))}, R_{char} = 50\Omega.
$$

Note that $S_{11}$ is a complex number. Fitting the measured $S_{11}$ using mBVD model yields: $R_m = 50\ \Omega$, $C_m = 0.0013\ pF$, and $L_m = 3000\ nH$, the fitting result is shown as the dashed line in Fig. S4(c)(d). The IDT conversion efficiency is defined as the power ratio of the power on mechanical lumped elements $P_m$ and the input power $P_{in}$. The input power that is transferred to $Y_m$ can be expressed as

$$
P_m = Re[V_{Y_m}^2 \cdot Y_m]
$$

where $V_{Y_m}$ is the voltage across the mechanical lumped elements, which can be calculated by analyzing the mBVD circuit in Fig. S4(a). The calculated electromechanical conversion efficiency reaches the highest value of 90% at resonance frequency 2.55 GHz in 4 K, as shown in Fig. S4(b).

### Characterizing $S_{21}$ signal

Generally, the time-gating signal processing is performed with following steps: (1) inverse Fourier transform (IFT) of the $S_{21}$ in frequency domain to the time domain, (2) filter out the RF crosstalk in time domain, and (3) transform the gated signal back to frequency domain. Fig. S5(a) shows the original $S_{11}$ signal taken from the VNA. The IFT of the $S_{21}$ signal is shown in Fig. S5(b). A strong signal at $t < 190$ ns can be seen; this is the free space radiation and the electronic background signal from the IDTs, so we filter out such a signal. Periodic sharp peaks that occur every 180 ns are also seen in the time-domain data, we attribute this to the reflection of bulk acoustic waves, which only contributes to an overall broadband background signal without distorting the main feature of the $S_{21}$ resonance in frequency domain. We can also clearly see two impulse responses at 210 ns and 400 ns.

The first pulse at 210 ns corresponds to the $L_2$ mode that is directly traveling through the acoustic waveguide, and the traveling path is shown in the inset of Fig. S5(d). The corresponding time-gated signal of this pulse is shown in Fig. S5(d). We can remove the free space RF crosstalk by filtering out the signal $< 190\ ns$. The calculated $L_2$ mode group velocity yields $v_{L_2} = 760/220\ \mu m/ns = 3454$ m/s, which is consistent with the simulation. The second pulse signal at 400 ns corresponds to the $L_2$ mode that is coupled out from the OMR. The traveling path of this pulse

(1388 $\mu$m) is shown in the inset of Fig. S5(c). The arrival time of the second pulse is consistent with the calculated acoustic velocity.

Finally, the signal is gated from $190 < t < 540$ ns, which corresponds to the superposition of the first and second pulse, as shown in Fig. S5(c). The interference of the both pulses will result in a free spectral range FSR = 5.5 MHz. Note that the Fourier transform is performed on a complex $S_{21}$ data. The similar time-gating technique is also described in other works [7, 8].

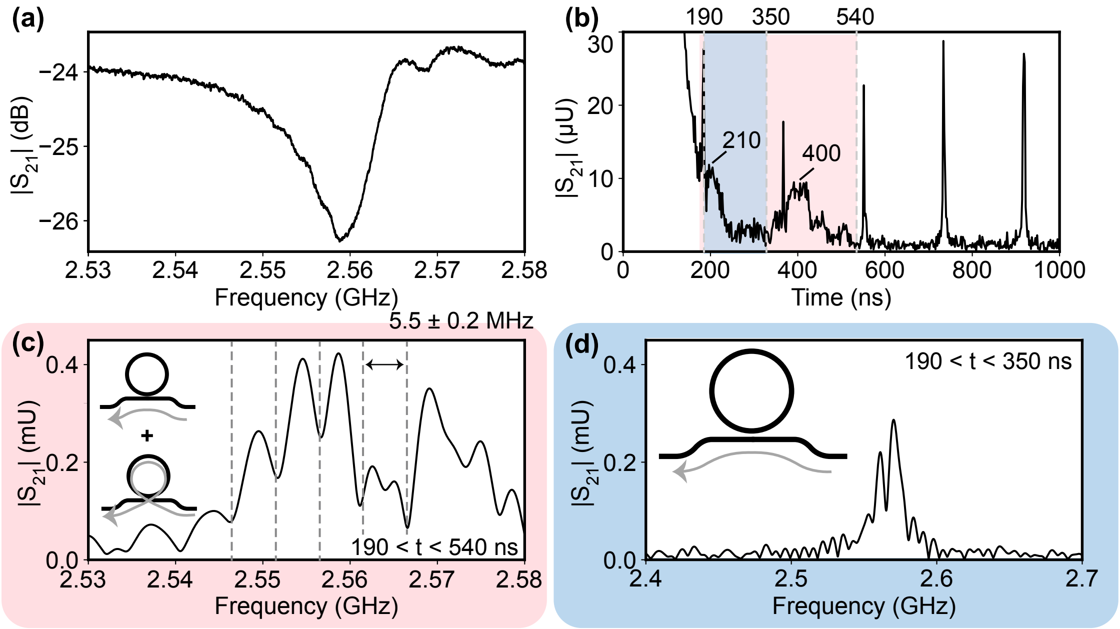


FIG. S5. **Time-gating signal processing of the acoustic transmission** $|S_{21}|$**.** **(a)** The unprocessed $|S_{21}|$ spectrum. **(b)** The $|S_{21}|$ in the time domain after Fourier transform. **(c)** The $|S_{21}|$ spectrum after time-gating from $190 < t < 540$ ns (the pink shaded window in b). The gray dashed line shows equal spacing of $5.5 \pm 0.2$ MHz. The inset in **(c)** shows the acoustic traveling path within the gated time window. **(d)** The $|S_{21}|$ spectrum after time-gating from $190 < t < 350$ ns (the blue shaded window in b). The inset shows the acoustic wave traveling path within the gated time window.

## Supplementary Note 5. PHASE-MATCHING CONDITIONS OF ALL POSSIBLE PROPAGATION DIRECTIONS OF THE OMR

The PMC measurement setup for all possible OMR configurations is shown in Fig. S6a. Both the optical and acoustic pump are fixed at the OMR resonance. Totally, four acoustic ports for input and output are available. In addition, two optical input ports and two optical output ports are available to select the circulation direction of the optical modes. Therefore, we can investigate all possible configurations of acoustic and optical input/output at all possible ports to characterize the time-reversal symmetry of the OMR. When the RF power is turned off, we observe a linear static $TE_2$ mode whose source is described in the main text, as shown as the black line in Fig. S6. When the RF is turned on, we observe strong $TE_2$ mode intensities increase for all counter-propagating acoustic and optical waves. This is the result of the dominant anti-Stokes-Brillouin process in the OMR, which is shown in the red lines in Fig. S6b-f. For the co-propagation cases, which are shown in Fig. S6g-k, we observe an optical output intensity retaining or drop in the optical spectrum analyzer (OSA), which can be attributed to the reduction of the undesired generated $TE_2$ mode because the out-scattering of $TE_0$ mode by the dominating co-propagating acoustic waves.

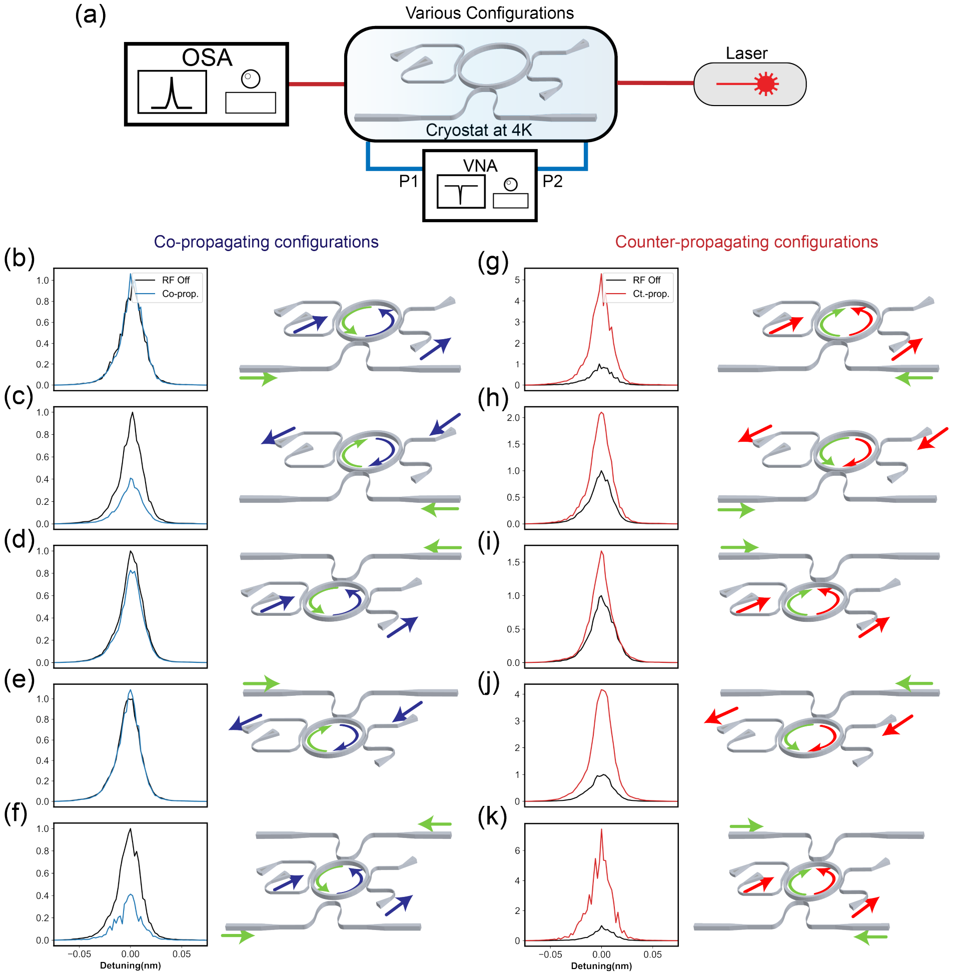


FIG. S6. **Phase-matching conditions of all co-propagating and counter-propagating configurations at cryogenic temperature. (a)** The measurement schematic of the PMC measurements. Both the laser and RF inputs are on resonance. Laser is fixed at resonance at 1571.67 nm and the RF driving at 2.56 GHz. The laser input is routed to the chip in the cryogenic probe station via the fiber probes and received at the OSA. **(b)-(f)** Shows the OSA spectrum with co-propagating configurations. **(g)-(k)** Shows OSA spectrum with counter-propagating configurations. Blue and red arrows show the co-propagating and counter-propagating optical waves, respectively. Green arrows show the acoustic wave propagation directions. The optical transmission for all the measurements shown here are normalized to the intensity when acoustic wave is turned off (black line).

## Supplementary Note 6. OMIC FABRICATION PROCESS FLOW

The OMIC fabrication process flow diagram is shown in Fig. S7.

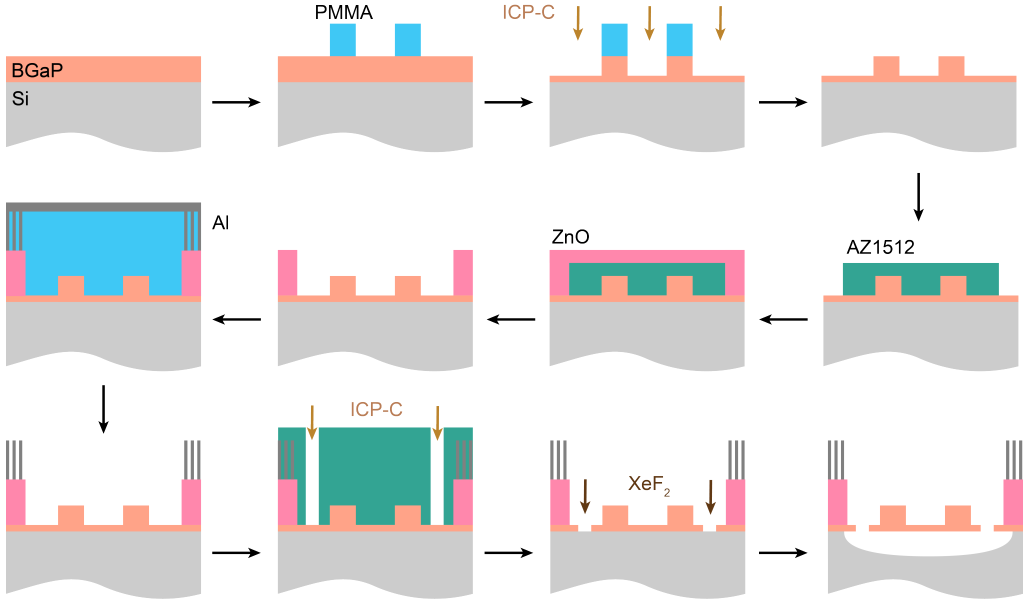


FIG. S7. **OMIC fabrication process flow.** We begin fabrication on a BGaP-on-Si chip and finished with a suspended BGaP film with ZnO and aluminum fingers on top. PMMA is patterned using EBL and AZ1512 photoresist is patterned using photolithography (Heidelberg DWL66+).

## Supplementary Note 7. DEVICE DESIGN PARAMETERS

More details of the device parameter are provided in the table. S3.

| OMIC device parameters | | | | |
|---|---|---|---|---|
| | Parameters | Unit | $TE_0$ | $TE_2$ |
| **Multi-mode OMR** | Wavenumber modes ($\beta_i$) | $\mu m^{-1}$ | 10.43 | 7.30 |
| | Wavenumber difference ($\beta_0 - \beta_2$) | $\mu m^{-1}$ | 3.13 | |
| | Effective mode index ($n_{eff}$) | | 2.558 | 1.784 |
| | Group index ($n_g$) | | 3.245 | 3.315 |
| | Hybrid $TE_0$-$TE_0$* ($\Delta n_{eff}$) | | 0.013 | |
| | Multi and single mode waveguide width (w) | $\mu m$ | 1.01 | 0.50 |
| | Ring diameter (D) | $\mu m$ | 200 | |
| | Ring total length ($l_{tot}$) | $\mu m$ | 628 | |
| | Optical coupling length ($L_{ii}$) | $\mu m$ | $L_{00} = 50$ | $L_{02} = 60$ |
| | Optical coupling gap width ($g_{ii}$) | $\mu m$ | $g_{00} = 0.07$ | $g_{02} = 0.20$ |
| | Waveguide to OMR coupling efficiency ($\eta_{ii}$) | % | $\eta_{00} = 90.0$ | $\eta_{02} = 3.0$ |
| | Single grating coupler efficiency ($\eta_{GC}$) | % | 3.0 | |
| | Optical FSR | $nm$ | 1.14 | 1.08 |
| | Parameters | Unit | $L_2$ | |
| **2nd-order Lamb mode ($L_2$)** | Acoustic wave central frequency ($\Omega/2\pi$) | GHz | 2.56 | |
| | Acoustic wavenumber ($\kappa$) | $\mu m^{-1}$ | 3.13 | |
| | Acoustic waveguide width ($w_a$) | $\mu m$ | 1.01 | |
| | Acoustic waveguide tapered length ($l_a$) | $\mu m$ | 100 | |
| | Acoustic Coupling length ($l_a$) | $\mu m$ | $l_a = \frac{2\pi}{\Delta K} = \frac{2\pi}{0.4} = 13$ | |
| | Acoustic Coupling gap width ($g_a$) | $\mu m$ | 0.2 | |
| | Simulated acoustic group velocity ($V_g$) | m/s | 3424 | |
| | Measured acoustic group velocity ($V_g$) | m/s | 3450 | |
| | Calculated Acoustic FSR | MHz | 5.4 | |
| | Measured Acoustic FSR | MHz | 5.5 | |
| | IDT pitch ($\Lambda$) | $\mu m$ | 2.0 | |
| | IDT aperture ($W$) | $\mu m$ | 15.0 | |
| | IDT efficiency ($\eta_a$) | % | 90.0 | |

TABLE S3. The phononic and photonic design parameters of the OMIC and OMR. *$\Delta n_{eff}$ is the effective refractive index difference in the $TE_0$-to-$TE_0$ coupling region that the hybrid mode has phase differences $\pi$, which helps us calculate the coupling length needed for critical coupling.

[1] S. G. Johnson, M. Ibanescu, M. A. Skorobogatiy, O. Weisberg, J. D. Joannopoulos, and Y. Fink, "Perturbation theory for maxwell's equations with shifting material boundaries," *Phys. Rev. E*, vol. 65, p. 066611, Jun 2002.
[2] B. G. Mytsyk, N. M. Demyanyshyn, and O. M. Sakharuk, "Elasto-optic effect anisotropy in gallium phosphide crystals," *Applied Optics*, vol. 54, no. 8546-8553, 2015.
[3] M. Wu, E. Zeuthen, K. C. Balram, and K. Srinivasan, "Microwave-to-optical transduction using a mechanical supermode for coupling piezoelectric and optomechanical resonators," *Phys. Rev. Appl.*, vol. 13, p. 014027, Jan 2020.
[4] X. Han, W. Fu, C.-L. Zou, L. Jiang, and H. X. Tang, "Microwave-optical quantum frequency conversion," *Optica*, vol. 8, pp. 1050–1064, Aug 2021.
[5] M. Aspelmeyer, T. J. Kippenberg, and F. Marquardt, "Cavity optomechanics," *Reviews of Modern Physics*, vol. 86, pp. 1391–1452, dec 2014.
[6] H. Li, Q. Liu, and M. Li, "Electromechanical Brillouin scattering in integrated planar photonics," *APL Photonics 4, 080802*, 2019.
[7] M. Bicer, S. Valle, J. Brown, M. Kuball, and K. C. Balram, "Gallium nitride phononic integrated circuits platform for ghz frequency acoustic wave devices," *Appl. Phys. Lett.*, vol. 120, no. 243502, 2022.
[8] F. M. Mayor, W. Jiang, C. J. Sarabalis, T. P. McKenna, J. D. Witmer, and A. H. Safavi-Naeini, "Gigahertz Phononic Integrated Circuits on Thin-Film Lithium Niobate on Sapphire," *Phys. Rev. Applied*, vol. 15, Jan 2021.
